# Radiotherapy for T1N0M0 Esophageal Cancer: Analyses of the Predictive Factors and the Role of Endoscopic Submucosal Dissection in the Local Control

**DOI:** 10.3390/cancers10080259

**Published:** 2018-08-03

**Authors:** Gen Suzuki, Hideya Yamazaki, Norihiro Aibe, Koji Masui, Daisuke Shimizu, Takuya Kimoto, Takeshi Nishimura, Akihiro Nakashima, Tadashi Takenaka, Osamu Dohi, Takeshi Ishikawa, Kei Yamada

**Affiliations:** 1 Department of Radiology, Kyoto Prefectural University Graduate School of Medical Science, 465 Kajiicho Kawaramachi Hirokoji, Kamigyo-ku, Kyoto 602-8566, Japan; yamahi@koto.kpu-m.ac.jp (H.Y.); a-ib-n24@koto.kpu-m.ac.jp (N.A.); mc0515kj@koto.kpu-m.ac.jp (K.M.); dshimizu@koto.kpu-m.ac.jp (D.S.); t-kimoto@koto.kpu-m.ac.jp (T.K.); Taknishi@koto.kpu-m.ac.jp (T.N.); anakashi@koto.kpu-m.ac.jp (A.N.); ttakenak@koto.kpu-m.ac.jp (T.T.); kyamada@koto.kpu-m.ac.jp (K.Y.); 2 Department of Gastroenterology and Hepatology Dermatology, Kyoto Prefectural University Graduate School of Medical Science, 465 Kajiicho Kawaramachi Hirokoji, Kamigyo-ku, Kyoto 602-8566, Japan; osamu-d@koto.kpu-m.ac.jp (O.D.); iskw-t@koto.kpu-m.ac.jp (T.I.)

**Keywords:** chemoradiation, endoscopic resection, ESD, stage I, prognostic factors, radiation, superficial esophageal cancer

## Abstract

Several therapeutic options are available for clinical T1N0M0 thoracic esophageal squamous cell carcinoma (stage I ESCC); however, the studies on the treatment results are limited. This study assessed the outcomes of stage I ESCC treated with radiotherapy (RT), determined predictive factors, and evaluated the benefits of endoscopic submucosal dissection (ESD) combined with RT. We retrospectively analyzed the data of 50 patients (41 men, 9 women; median age, 66 years) with stage I ESCC treated with RT. The median total irradiation dose was 50 Gy. Elective nodal irradiation (ENI) was performed in 17 patients and ESD in 29 patients (ESD group). Forty-six patients concurrently received chemotherapy with RT. The median tumor length of ESD and non-ESD groups was 2.3 and 5 cm, respectively. The median follow-up was 33 months. The 3-year overall survival, disease-free survival (DFS), and local control (LC) rates were 77.3%, 61.1%, and 88.1%, respectively. Grade 3 adverse events occurred in 14 patients. T stage and tumor length were significant prognostic factors for 3-year DFS and 3-year LC, respectively. ESD appeared to be an important prognostic factor for LC. ENI and total irradiation dose above 50.4 Gy were not predictive factors. Our findings might help in treatment decisions for stage I ESCC.

## 1. Introduction

The epidemiology of esophageal cancer has evolved over the past 2 decades. One of the most significant changes is the shift in stage distribution. The incidence of superficial esophageal cancer that invades up to the submucosa is increasing, particularly in Asian countries where endoscopic screening for cancers of the upper digestive tract is common [1,2]. According to the Comprehensive Registry of Esophageal Cancer in Japan, the incidence of clinical stage 0/1 cancer increased from 23.1% to 30.8% of all patients from 1999–2009 [3].

The management of esophageal cancer has also evolved over time. Esophagectomy with extended lymph node dissection has historically been the standard approach for T1N0M0 thoracic esophageal squamous cell cancer (stage I ESCC) with deep mucosal or submucosal involvement. Although the 3-year survival rate of surgically treated patients with stage I ESCC has been reported to be >80%, the disadvantages include a substantial risk of major surgical complications, a minor but real risk of perioperative death, a recovery period of several months, and the potential for long-term swallowing problems [4,5].

With the development of endoscopic equipment, the use of endoscopic resection (ER) for treating early-stage esophageal cancer has increased [6,7]. However, this approach is only appropriate for patients with a very low risk of lymph node metastasis. Currently, the candidates for radiotherapy (RT) or chemoradiotherapy (CRT) mainly include patients with medically difficult cancers and those who refuse to undergo endoscopic/surgical resection. Although no randomized clinical trial has compared definitive CRT and surgery for resectable stage I ESCC, the efficacy of CRT has recently been suggested in a few studies [8,9].

One of the main limitations of definitive CRT is local failure. Combined treatment involving ER and RT/CRT is considered as one of the treatment strategies for stage I ESCC patients with a high risk of recurrence [10]. The number of reports on the results of RT/CRT for stage I ESCC is limited, and furthermore, the necessity of elective nodal irradiation (ENI) and combined treatment involving endoscopic submucosal dissection (ESD) for stage I ESCC remains unclear.

The present study aimed to assess the outcomes of stage I ESCC patients treated with RT, determine the predictive factors, and evaluate the benefits of ESD combined with RT.

## 2. Results

### 2.1. Patient Characteristics

There were 50 patients with esophageal cancer who enrolled in this study. Among them, 29 (58%) patients underwent ESD followed by RT/CRT (ESD group). The background and treatments of the ESD and non-ESD groups are summarized in Table 1. The median age of the ESD group was significantly lower than that of the non-ESD group (*p* = 0.018). No significant between-group difference was observed in the location and number of the tumor, T stage, the use of concurrent chemotherapy, and radiation field. The median tumor lengths of the ESD and non-ESD groups were 2.3 cm (range, 1–10 cm) and 5 cm (range, 1–20 cm), respectively, which was statistically significant (*p* = 0.017). The total radiation dose was ≤50.4 Gy in 26 patients (90%) in the ESD group, whereas nine of the 21 patients (43%) in the non-ESD group received >50.4 Gy (*p* = 0.02). Chemotherapy was concurrently administered with RT in 46 (92%) patients (28 in the ESD group and 18 in the non-ESD group). Regarding the regimen of chemotherapy, 36 received 2 cycles of 5-fluorouracil and cisplatin (20 in the ESD group and 16 in the non-ESD group), 7 received 1 cycle of 5-fluorouracil and cisplatin (6 in the ESD group and 1 in the non-ESD group), 2 in the ESD group received 2 cycles of 5-fluorouracil and nedaplatin, and 1 in the non-ESD group received S-1 (orally administered twice daily for 14 days every 3 weeks). The patient and tumor characteristics are summarized in Table 1. Histopathological findings of the 29 patients who underwent ESD are summarized in Table 2. Regarding the depth of the invasion, the M3, SM1, SM2, and SM3 cancers were diagnosed in 8, 4, 16, and 1 patients, respectively. The rate of microscopic incomplete resection (R1 resection) after ESD was 34% (10/29 patients). 

### 2.2. Treatment Outcomes

**Figure 1:**
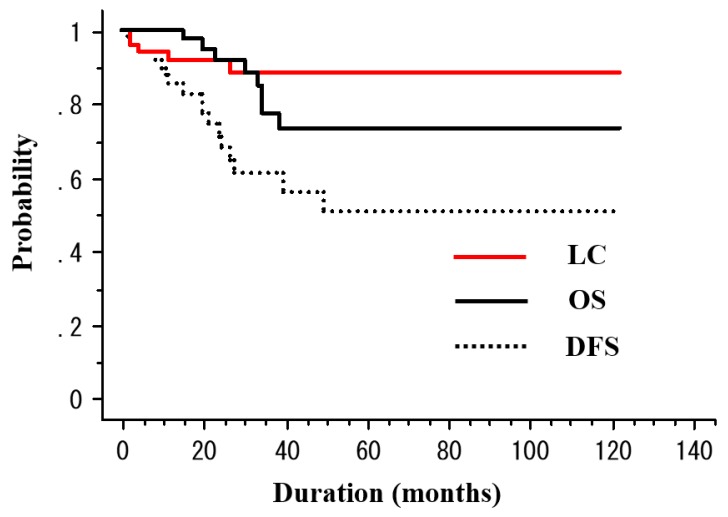
The overall survival (OS), disease-free survival (DFS), and local control (LC) rates for patients with T1N0M0 esophageal cancer treated with radiotherapy.

**Figure 2:**
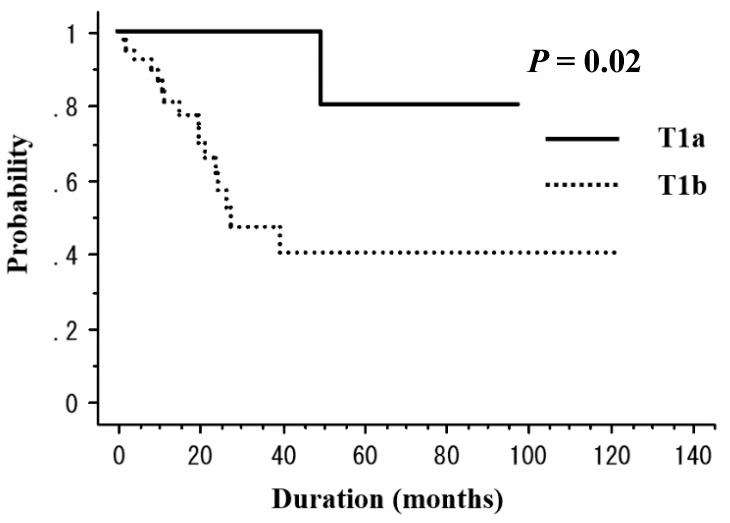
The disease-free survival (DFS) rate according to the T stage. The DFS rates are 100% in patients with T1a tumors and 47.1% in those with T1b tumors at 3 years (*p* = 0.02).

**Figure 3:**
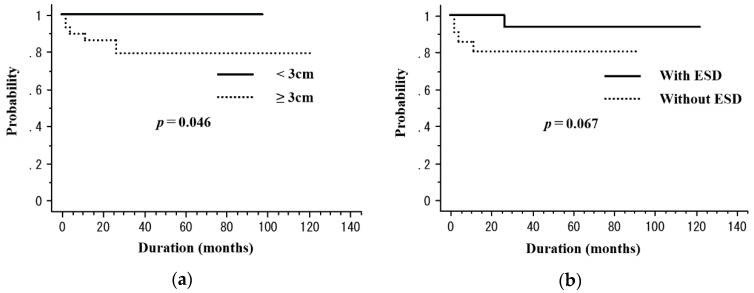
The local control (LC) rate according to the tumor length and the endoscopic submucosal dissection (ESD) treatment. (**a**) The LC rates are 100% in patients with tumors <3 cm and 79.3% in those with tumors ≥3 cm at 3 years (*p* = 0.046). (**b**) The LC rates are 93.3% in patients who received ESD treatment and 80.4% in those who did not receive ESD treatment (*p* = 0.067).

The median observation period was 33 months (range, 6–122 months). RT was completed in all patients. Among the 50 patients, 5 (10%) died of esophageal cancer and 3 (6%) died of other causes (sepsis with deep vein thrombosis, *n* = 1; oropharyngeal cancer, *n* = 1; congestive heart failure, *n* = 1) throughout the study period. For the patient who died of congestive heart failure, treatment-related death was unlikely because his tumor was located in the upper thoracic and treated with non-ENI. Recurrence was observed in 15 (30%) patients (local recurrence in 4 (8%), regional lymph node metastasis in 4 [8%], distant metastasis in 2 [4%], and metachronous esophageal lesions in 5 (10%)). After the identification of recurrence, 5 patients underwent salvage ESD, 6 received chemotherapy, 1 received CRT, and 1 underwent argon plasma coagulation (Table 3). The 3-year rates of overall survival (OS), disease-free survival (DFS), and local control (LC) were 76.8%, 46.9%, and 64.1%, respectively (Figure 1). Table 4 presents the results of the univariate analysis for DFS and LC. The T stage (T1a vs. T1b, 100% vs. 47.1%, *p* = 0.02) was a significant prognostic factor for 3-year DFS (Figure 2). In addition, the tumor length (<3 cm vs. ≥3 cm, 100% vs. 79.3%, *p* = 0.046) was a significant prognostic factor for 3-year LC (Figure 3a). ESD treatment (yes vs. no, 93.3% vs. 80.4%, *p* = 0.068) appeared to be an important prognostic factor for LC; however, there was no statistical significance (Figure 3b).

### 2.3. Toxicity

An acute adverse events (AE) grade that was ≥3 occurred in 14 (28%) patients (grade 3 leukopenia in 10 (20%), grade 3 anorexia in 3 (6%), and grade 3 esophagitis in 1 (2%)). None of the patients experienced treatment interruption associated with acute toxicities lasting over 1 week. Additionally, none of the patients had a toxicity grade that was ≥4.

A late AE grade that was ≥2 was noted in 6 (12%) patients (grade 2 esophageal strictures in 4 (8%) and grade 2 pericardial effusion without any symptoms in 2 (4%)) during the follow-up (Table 5). A late AE grade that was ≥3 was not identified.

## 3. Discussion

Esophagectomy is the standard treatment for stage I ESCC, and CRT is considered the best alternative treatment to esophagectomy. In the JCOG9708 trial, Kato et al. included 72 patients who did not meet the indications for ER and were treated with CRT at 60 Gy with concurrent 5-fluorouracil and cisplatin [8]. In this trial, a complete response was observed in 87.5% of the patients, with a 4-year OS rate of 80.5% and a relapse-free survival rate of 68.0%. In a more recent retrospective study by Koide et al., 123 patients with stage I ESCC were treated with RT or CRT. In their study, the 5-year OS rate was 77% [9]. These findings indicate that the survival rates after CRT are comparable to those after surgery for stage I ESCC, with approximately 80% of the patients experiencing long-term survival [3]. Our study identified a 3-year OS rate of 76.8%, which is slightly lower than that reported in the above-mentioned studies.

There are few reports on stage I ESCC treated with definitive RT/CRT. Furthermore, clinical trials often fail to include elderly patients. To our knowledge, there have been few reports on CRT for stage I ESCC in elderly patients. As elderly patients might not be able to undergo esophagectomy because of comorbidities, RT is considered as a reasonable alternative. More than half of the patients treated in our study were elderly patients (>70 years), and this might explain the worse treatment outcomes in our study than in previous reports. We did not find statistically significant differences in the DFS and LC rates between elderly patients and other patients. In our study, RT was effective and safe for stage I ESCC, even in elderly patients, and therefore, it can be applied in routine clinical practice.

One of the major drawbacks of definitive RT is a high incidence of local failure [11,12]. Even with superficial tumors, the local recurrence rate can be up to 30% [8,9,13]. Some patients with local recurrence require an esophagectomy, which is generally associated with high morbidity and mortality [14].

In our study, tumor length (>3 cm vs. ≤3 cm) was found to be a prognostic factor for LC. Koide et al. reported that the unfavorable factors for LC in patients with stage I ESCC treated with CRT were a large tumor length (>3 cm), male sex, a large tumor circumference (>1/2), and T1b stage [9]. Furthermore, in their report, large tumor length (>3 cm) was found to be a significant prognostic factor for not only LC but also DFS. Thus, the tumor length can be considered an important factor for LC, and it may help in treatment decisions for stage I ESCC.

It is unclear whether the addition of ER to RT/CRT for stage I ESCC can improve clinical outcomes [10]. In our study, ESD showed a close association with the LC rate; however, the relationship did not reach statistical significance. A recent retrospective study by Kawaguchi et al. compared the treatment outcomes of ESD followed by CRT and those of definitive CRT [13]. The authors found that the 3-year OS, loco-regional control, and LC were better with combined ESD and CRT; however, the findings were not statistically significant. Hamada et al. reported that ER combined with CRT was a favorable treatment option for patients with stage I ESCC, as local recurrence occurred in only 2 of 66 (3%) patients and the 1-, 3-, and 5-year local recurrence rates were 0.0%, 1.5%, and 1.5%, respectively [15]. Uchinami et al. reported similar results with combined treatment and mentioned significance with regard to the LC rate [16].

As mentioned above, data from published studies illustrate that LC might be better with combined ER and CRT than CRT alone. However, these were not randomized studies, and they had selection bias. Indeed, most patients treated with definitive CRT in the Japanese Phase II trial (JCOG9708) did not fulfill the indications for ER as they had aggressive disease. In the study by Kawaguchi et al., 80% of the patients (25/31) who received definite CRT showed massive tumor extension in the circumferential or longitudinal direction on endoscopic ultrasound-based diagnosis [13]. Therefore, patients treated with definitive CRT may have larger tumors and, hence, poor outcomes. In fact, in our study, the tumor length of the non-ESD group was also significantly larger than that of the ESD group (Table 1). Combined ESD and CRT might be useful for LC in stage I ESCC; however, in our study, it was not beneficial for improving DFS. A phase II study is ongoing in Japan to evaluate the efficacy and safety of ESD followed by CRT for clinical stage I (T1bN0M0) esophageal cancer [17], and the results are expected in the near future.

Esophageal strictures are common AE after ESD, and the tumor circumferential extension was reported to be a risk factor for postoperative strictures [18,19]. Treatment-related esophageal strictures were observed in 4 patients (8%) in the present study, and the patients were managed with medication and endoscopic balloon dilatation. Of these 4 patients, 3 had undergone ESD followed by CRT. In all patients with esophageal strictures, the tumor size was 5 cm or larger. Therefore, not only tumor circumferential extension but tumor size might also be an important risk factor for esophageal strictures after treatment.

ENI and an irradiation dose above 50.4 Gy did not improve clinical outcomes in our study. With regard to the RT field, it is controversial whether ENI is necessary for stage I ESCC. Yamashita et al. retrospectively analyzed 126 patients treated with CRT and concluded that ENI was effective for preventing regional lymph node failure as none of the patients experienced elective node failure, without any other recurrence site [20]. Additionally, Onozawa et al. analyzed 102 patients and reported that ENI was effective for preventing regional lymph node recurrence [21]. In contrast, Zhao et al. analyzed 53 patients treated with RT and concluded that the omission of ENI was not associated with significant regional lymph node failure [22].

An important issue with these previous studies is that they included ESCC patients at various stages, and adequate evidence is not available for patients with stage I ECSS. Moreover, in these previous studies, chemotherapy might have contributed to the prevention of recurrence. Uchinami et al. retrospectively analyzed 90 patients with clinical stage I ESCC treated with RT or CRT (39 with ENI and 51 without ENI) and reported that ENI was not an independent prognostic factor for DFS [16]. The results of our study are comparable to their clinical outcomes, and the findings indicate that ENI may not be required for stage I ESCC.

The present study had several limitations, including a retrospective design, a small sample size, and a short follow-up period, which may have limited the statistical power. Furthermore, the treatment strategies for the patients slightly differed over time. A prospective, randomized, controlled study with a large number of patients and long follow-up period is necessary for the selection of the most appropriate treatment option for stage I ESCC.

## 4. Materials and Methods

### 4.1. Study Design and Population

The study included 50 patients with histopathologically confirmed stage I ESCC who received external-beam RT at our hospital between June 2009 and September 2017. Curative ESD was performed in 29 of these patients before RT/CRT. In patients who did not undergo ESD, ESCC was diagnosed as clinical T1 cancer using magnifying endoscopy or endoscopic ultrasonography. At our hospital, almost all clinical stage I ESCC patients are advised to undergo surgery or ESD because of the favorable survival rate and good safety of these approaches. All patients in the present study were medically ineligible to undergo esophagectomy or refused to undergo it. Lymph node or distant metastasis was ruled out with contrast-enhanced computed tomography (CT) from the neck to the abdomen and positron emission tomography/CT of the whole body. Treatment was decided by a tumor board that included surgeons, endoscopists, medical oncologists, and radiation oncologists. The study was approved by the institutional review board of the Kyoto Prefectural University of Medicine (approval number: ERB-C-1179). Written informed consent was obtained from each patient prior to treatment.

### 4.2. Treatment

In the 29 patients who underwent curative ESD, the ESD specimen was examined pathologically for histological type, depth of tumor invasion, resection margin, and lymphatic and venous tumor invasion. Based on the depth of invasion, mucosal lesions were classified as M1 for intraepithelial carcinomas, M2 for tumors invading the lamina propria, or M3 for tumors in contact with or infiltrating the muscularis mucosae; submucosal lesions were classified as SM1 for tumors invading the more superficial layer of the submucosa (corresponding to one-third of its thickness), SM2 for tumors invading the middle-third, and SM3 for tumors invading the deeper submucosal layer [23]. In our institution, all patients, except those having a pT1a tumor with a negative resection margin and no lymphovascular invasion, receive additional RT after ESD.

All study patients underwent CT simulations before RT. The gross tumor volume (GTV) or tumor bed after ESD was marked with a clip before planning CT. The patients received 3-dimensional conformal RT (1.8–2 Gy/day for 5 days a week) with a linear accelerator (6 or 10 MV). The bilateral supraclavicular, periesophageal, mediastinal, and perigastric lymph node areas were considered as regional lymph node areas. Prophylactic RT involving these lymph node areas was considered as ENI. The GTV was expanded to the clinical tumor volume (CTV) by extending the margin 2–3 cm superiorly and inferiorly and 0.5 cm laterally. The CTV included the prophylactic regional lymph nodes along with the primary tumor or tumor bed in ENI patients, while it included the GTV with an optional part of the regional lymph nodes in non-ENI patients. The planning target volume was defined as the CTV plus a 1-cm margin in all directions in the initial and boost plans. In non-ENI patients, a dose of 39.6–40 Gy in 20–22 fractions was delivered with anterior/posterior opposed portals or anterior/posterior and oblique four-portals.

After the initial plan was completed, a boost of 9–20 Gy was delivered to the primary tumor. In patients who underwent ESD, the boost was delivered to the tumor bed only when there was a positive resection margin. Treatment fields were adjusted using a multileaf collimator in order to reduce the maximal dose delivered to the spinal cord to <40 Gy.

The most common regimen of chemotherapy was 2 cycles of 5-fluorouracil and cisplatin. Between 2009 and 2013, chemotherapy involved administration of 5-fluorouracil (700 mg/m^2^) intravenously on days 1–4 and cisplatin (70 mg/m^2^) intravenously on day 1 every 4 weeks. From January 2014, the doses of 5-fluorouracil and cisplatin were increased to 1000 mg/m^2^ and 75 mg/m^2^, respectively. The use of chemotherapy and the regimen were determined by physicians according to each patient’s general condition, clinical course, and function of organs, such as the kidneys, liver, heart, and bone marrow.

### 4.3. Follow-Up and Evaluation

All patients were followed-up for the detection of local recurrence or distant metastasis every 3–4 months during the first 2 years and every 6 months thereafter. The follow-ups involved blood tests, upper gastrointestinal endoscopy with iodine staining, and CT of the neck, chest, and abdomen. Follow-up data were obtained from electronic medical records.

### 4.4. Statistical Analysis

Comparisons of patient and tumor characteristics were performed with the chi-square test, 2-sample t-tests, or Wilcoxon rank sum tests. The OS was measured from the date of treatment initiation to the date of last follow-up or death from any cause. The DFS was measured from the date of treatment initiation to the date of first observation of any recurrence or death from any cause. The LC was measured from the date of treatment initiation to the date of local recurrence of the primary tumor. Residual tumors after RT were considered to indicate treatment failure. The OS, DFS, and LC rates were calculated using Kaplan–Meier estimates. In univariate analysis, age (≤70 years vs. >70 years), sex (male vs. female), Eastern Cooperative Oncology Group Performance Status (ECOG PS) (0 vs. ≥1), T stage (T1a vs. T1b), tumor number (1 vs. ≥2), tumor length (<3 cm vs. ≥3 cm), irradiation dose (≤50.4 Gy vs. >50.4 Gy), radiation field (ENI vs. non-ENI), and ESD treatment (yes vs. no) were assessed using the log-rank test. Toxicities were scored according to the National Cancer Institute Common Terminology Criteria for Adverse Events (NCI-CTCAE) version 4.0 [24]. A *P* value of <0.05 was considered to indicate statistical significance. All statistical analyses were performed using EZR (Saitama Medical Center, Jichi Medical University, Saitama, Japan), which is a graphical user interface for R (The R Foundation for Statistical Computing, Vienna, Austria, version 2.13.0). More precisely, it is a modified version of R commander (version 1.6–3) that was designed to add statistical functions frequently used in biostatistics [25].

## 5. Conclusions

T stage is a predictive factor for DFS, while tumor length is a predictive factor for LC. Additionally, the combined ESD and RT treatment might improve LC. ENI and a total irradiation dose above 50.4 Gy have no significant impact on treatment outcomes. Our findings might help in treatment decisions for clinical stage I ESCC.

## Figures and Tables

**Table 1:** The patient and tumor characteristics.

	ESD ^2^ Group	Non-ESD Group	*p* Value
Characteristic	(*n* = 29)	(*n* = 21)	
Age (years)			
Median (range)	68 (50–82)	75 (59–87)	0.018
Sex, n			
Male	24	17	0.99
Female	5	4	
Performance status, n			
0	25	16	0.59
≥1	4	5	
Main tumor location, n			
Upper thorax	4	4	0.44
Middle thorax	13	10	
Lower thorax	12	7	
T Stage, n			
T1a	8	3	0.44
T1b	21	18	
Tumor length (cm)			
Median (range)	2.3 (1–10)	5 (1–20)	0.017
Tumor number, n			
1	5	6	0.34
≥2	24	15	
Concurrent chemotherapy, n			
yes	28	18	0.39
no	1	3	
Radiation field, n			
ENI ^1^	13	4	0.11
Non-ENI	16	17	
Total radiation dose, n			
≤50.4 Gy	26	12	0.02
>50.4 Gy	3	9	

^1^ ENI: Elective nodal irradiation, ^2^ ESD: Endoscopic submucosal dissection.

**Table 2:** The histopathological findings in the ESD group.

	Number (%)	Depth of Invasion			
	All Patients	M3	SM1	SM2	SM3
Number	29	8	4	16	1
Resection status					
R0 ^1^ resection	19 (66%)	6	2	11	0
R1 ^2^ resection	10 (34%)	2	2	5	1
Lymphovascular invasion					
Positive	20 (69%)	5	3	11	1
Negative	9 (31%)	3	1	5	0
Poorly differentiated histology					
Yes	4 (14%)	1	0	3	0
No	25 (86%)	7	4	13	1

^1^ R0: No cancer at resection margins, ^2^ R1: Microscopic residual cancer.

**Table 3:** The summary of recurrent cases.

Age	Sex	PS ^12^	T Stage	Tumor Length (cm)	RT ^13^ Field	RT Dose (Gy)	ESD ^8^	Resection Status	Lymphovasucular Invasion	Depth of Invasion	Poorly Differentiated Histology	CCRT ^4^	Months to Disease Recurrence	Recurrence Site	Salvage Therapy	Status at Last Follow-Up
68	Male	0	T1b	2	ENI ^7^	40	+	R0 ^14^	+	SM1	–	+	11	Metachronous esohageal lesion (IF ^9^)	ESD	ANED ^1^ 20 m ^11^
60	Male	0	T1b	8	ENI	50	+	R1 ^15^	+	SM3	–	+	24	Distance	chemo	DID ^5^ 33 m
69	Male	1	T1b	2.1	ENI	40	+	R0	+	SM2	–	+	10	Metachronous esohageal lesion (OF ^10^)	ESD	ANED 45 m
59	Male	0	T1a	3.4	Non-ENI	50.4	+	R0	–	M3	+	+	50	Metachronous esohageal lesion (OF)	ESD	ANED 79 m
61	Male	0	T1b	1	Non-ENI	50	+	R0	+	SM2	–	+	40	Regional (OF)	CRT	ANED 98 m
75	Male	1	T1b	2.5	Non-ENI	60	+	R1	+	SM2	+	+	9	Regional (OF)	chemo	AWD ^3^ 21 m
65	Male	0	T1b	10	Non-ENI	40	+	R0	–	SM2	–	+	27	Local	APC ^2^	ANED 112 m
57	Male	0	T1b	1	Non-ENI	60	+	R1	+	SM2	–	–	24	Regional (IF)	chemo	DID 34 m
86	Male	0	T1b	6	Non-ENI	42	–					–	2	Local	chemo	DID 31 m
61	Male	0	T1b	5	ENI	50.4	–					+	4	Metachronous esohageal lesion (OF)	ESD	ANED 65 m
71	Male	0	T1b	6	Non-ENI	50.4	–					+	12	Local	chemo	DID 34 m
75	Male	0	T1b	1.5	Non-ENI	60	–					+	20	Metachronous esohageal lesion (OF)	ESD	ANED 92 m
83	Female	2	T1b	3	Non-ENI	50	–					+	2	Local	no	AWD 39 m
80	Male	1	T1b	8	Non-ENI	54	–					+	22	Regional (OF)	no	DOD ^6^ 23 m
70	Male	0	T1b	1.5	Non-ENI	60	–					+	28	Distance	chemo	DID 38 m

^1^ ANED: Alive with no evidence of disease, ^2^ APC: Argon plasma coagulation, ^3^ AWD: Alive with disease, ^4^ CCRT: Concurrent chemoradiotherapy; ^5^ DID: Died of inter-current disease, ^6^ DOD: Died of the disease, ^7^ ENI: Elective nodal irradiation, ^8^ ESD: Endoscopic submucosal dissection, ^9^ IF: In the irradiation field, ^10^ OF: Out of the irradiation field, ^11^ m: Months, ^12^ PS: Performance status, ^13^ RT: Radiotherapy, ^14^ R0: No cancer at resection margins, ^15^ R1: Microscopic residual cancer.

**Table 4:** The univariate analysis for the disease-free survival and local control rates.

	*N*	3-Year DFS ^1^ (%)	*p* Value	3-Year LC ^4^ (%)	*p* Value
Age (years)					
≤70	24	62.2	0.99	87.9	0.63
>70	26	61.1		89	
Sex					
Male	41	57.8	0.38	88.3	0.82
Female	9	90		90	
PS ^5^					
0	41	66.1	0.08	88.1	0.86
≥1	9	38.1		90	
T stage					
T1a	11	100	0.02	100	0.18
T1b	39	47.1		83.8	
Tumor number					
1	39	67.7	0.43	87.7	0.89
≥2	11	39		100	
Tumor length					
<3 cm	21	55.1	0.55	100	0.046
≥3 cm	29	65.1		79.3	
Radiation dose					
≤50.4 Gy	38	63.9	0.94	84.5	0.19
>50.4 Gy	12	53.9		100	
Radiation field					
ENI ^2^	17	69.9	0.65	100	0.53
Non-ENI	33	58.5		85.6	
ESD ^3^					
Yes	29	70	0.37	93.3	0.067
No	21	52.4		80.4	

^1^ DFS: Disease-free survival, ^2^ ENI: Elective nodal irradiation, ^3^ ESD: Endoscopic submucosal dissection, ^4^ LC: Local control, ^5^ PS: Performance status.

**Table 5:** The summary of late toxicities of a grade ≥2 in the study patients.

Age (Years)	Sex	Adverse Event	Grade	RT Field	ESD ^3^	CRT ^1^	Tumor Length (cm)	Tumor Circumferential Extension	RT ^4^ Dose (Gy)
80	Female	Pericardial effusion	2	Non-ENI ^2^	−	+	20	Entire	50
70	Female	Pericardial effusion	2	Non-ENI	−	+	15	Entire	60
54	Female	Esophageal strictures	2	ENI	+	+	5	1/2	40
65	Male	Esophageal strictures	2	ENI	+	+	7.4	2/3	40
60	Male	Esophageal strictures	2	ENI	+	+	8	2/3	50
59	Male	Esophageal strictures	2	Non-ENI	−	+	14	Entire	50.4

^1^ CRT: Concurrent chemoradiation, ^2^ ENI: Elective nodal irradiation, ^3^ ESD: Endoscopic submucosal dissection, ^4^ RT: Radiotherapy.
